# Streamlined process for effective and strand-selective mitochondrial base editing using mitoBEs

**DOI:** 10.52601/bpr.2024.240010

**Published:** 2024-08-31

**Authors:** Xiaoxue Zhang, Zongyi Yi, Wei Tang, Wensheng Wei

**Affiliations:** 1 Changping Laboratory, Beijing 102206, China; 2 Biomedical Pioneering Innovation Center, Peking-Tsinghua Center for Life Sciences, Peking University Genome Editing Research Center, State Key Laboratory of Protein and Plant Gene Research, School of Life Sciences, Peking University, Beijing 100871, China; 3 Academy for Advanced Interdisciplinary Studies, Peking University, Beijing 100871, China

**Keywords:** Mitochondrial DNA base editors (mitoBEs), Transcription-activator-like effector (TALE), Deaminase, Nickase

## Abstract

Mitochondrial base editing tools hold great promise for the investigation and treatment of mitochondrial diseases. Mitochondrial DNA base editors (mitoBEs) integrate a programmable transcription-activator-like effector (TALE) protein with single-stranded DNA deaminase (TadA8e-V106W, APOBEC1, *etc*.) and nickase (MutH, Nt.BspD6I(C), *etc*.) to achieve heightened precision and efficiency in mitochondrial base editing. This innovative mitochondrial base editing tool exhibits a number of advantages, including strand-selectivity for editing, high efficiency, and the capacity to perform diverse types of base editing on the mitochondrial genome by employing various deaminases. In this context, we provide a detailed experimental protocol for mitoBEs to assist others in achieving proficient mitochondrial base editing.

## INTRODUCTION

Numerous mutations in the nuclear genome can cause diseases, and in addition to the nuclear genome, mitochondria possess their own genome as semi-autonomous organelles within human cells. Mutations in the mitochondrial genome can also cause various diseases affecting multiple tissues and organs such as the heart, digestive system, nervous system, eyes, and ears (Vafai and Mootha 2012). Notable mitochondrial diseases include Leber hereditary optic neuropathy (LHON), mitochondrial encephalopathy, lactic acidosis and stroke-like episodes (MELAS) syndrome, and Leigh syndrome. LHON typically manifests in young males with progressive visual loss due to optic neuropathy, while MELAS and Leigh syndrome, more common in children, primarily impact the nervous system and muscles. Currently, there are 97 confirmed mutations associated with mitochondrial diseases, with 93 being point mutations and 4 indels, where point mutations account for 95% (MITOMAP). It is noteworthy that mitochondrial DNA (mtDNA) usually exists in multiple copies, and symptoms generally manifest when the mutation load reaches approximately 60% (Gammage *et al*. 2018).

Mitochondrial disease treatment options currently include mitochondrial DNA replacement therapy, involving the removal of the nucleus from healthy cells, leaving the cytoplasm, and transferring the nuclei of diseased cells to replace them with healthy mitochondria (Adashi *et al*. 2021). This treatment gained approval in the UK in 2015. However, a drawback of this method is the retention of some mutant mitochondria during the nuclear transfer process, accounting for about 2.37% to 9.23% (Adashi *et al*. 2021). The full impact of these remaining mitochondria on the organism is not yet fully understood.

An alternative approach is gene editing therapy, leveraging the DNA-binding capabilities of zinc-finger proteins and TALE proteins. Researchers have fused these proteins with the nuclease FokI and mitochondrial targeting sequence (MTS) to create gene editing tools known as mitochondrially targeted zinc-finger nucleases (mitoZFNs) and mitochondrially targeted transcription activator-like effector nucleases (mitoTALENs) (Gaj *et al*. 2013). These tools can selectively cleave DNA, leading to double-strand breaks and subsequent degradation of mtDNA (Peeva *et al*. 2018). This method aims to specifically target mutated mtDNA for degradation, leading to an increase in the proportion of wild-type mtDNA and achieving the intended treatment goals (Silva-Pinheiro and Minczuk 2022). However, this approach is not suitable for homozygous mutation-related diseases, as complete degradation of all mtDNA is not feasible. Additionally, this method lacks the capability to make specific sequence changes to mtDNA. Given that 95% of mtDNA mutations associated with mitochondrial diseases are single-base mutations, the development of mitochondrial DNA base editing tools becomes crucial for more precise and effective treatment strategies.

While current base editors for nuclear genomes rely on the CRISPR system, its application for mitochondrial gene editing is hindered by low efficiency. The physical and chemical characteristics of the mitochondrial inner membrane prevent efficient entry of the gRNA required for the CRISPR system (Gammage *et al*. 2018). Therefore, an all-protein system is needed for efficient mitochondrial base editing. The discovery of DddA, a double-stranded DNA deaminase, paved the way for the fusion of DddA with TALE proteins, resulting in the development of mitochondrial cytosine base editors (DdCBEs) (Mok *et al*. 2020). Subsequently, the deaminase active center of DddA was deactivated, combined with the adenine deaminase TadA8e, leading to mitochondrial adenine base editors known as TALEDs (Cho *et al*. 2022). While DdCBEs and TALEDs have TALE-dependent and TALE-independent off-target effects, their editing is not precise, affecting both strands in the editing window. The preference of DddA for 5'-TC sequences limits its efficiency in editing 5'-GC sequences (Mok *et al*. 2022). Homologous proteins of DddA were discovered, creating new mitochondrial cytosine base editing tools without sequence preference, albeit with similar imprecision (Guo *et al*. 2023; Mi *et al*. 2023).

In addition to utilizing double-stranded DNA deaminase, engineered single-stranded DNA deaminase has gained widespread application, exemplified by the working principle of CRISPR-based base editor. The underlying concept involves sgRNA guiding the Cas protein to the target DNA, forming an R-loop structure that exposes the single-stranded structure of the sgRNA non-targeting strand. Single-stranded DNA deaminase then performs the deamination of single-stranded DNA, converting cytosine to uracil or adenine to inosine. During DNA repair or replication, uracil and inosine are interpreted as thymine and guanine, respectively, thereby achieving C-to-T and A-to-G base editing (Gaudelli *et al*. 2017; Komor *et al*. 2016). The substrates for these DNA deaminase enzymes are all single-stranded DNA. Prior investigations attempted to directly employ the CRISPR system for mitochondrial gene editing, however, the outcomes were suboptimal, with editing efficiencies consistently below 1% (Hussain *et al*. 2021; Jo *et al*. 2015; Wang *et al*. 2021).

**Figure 1:**
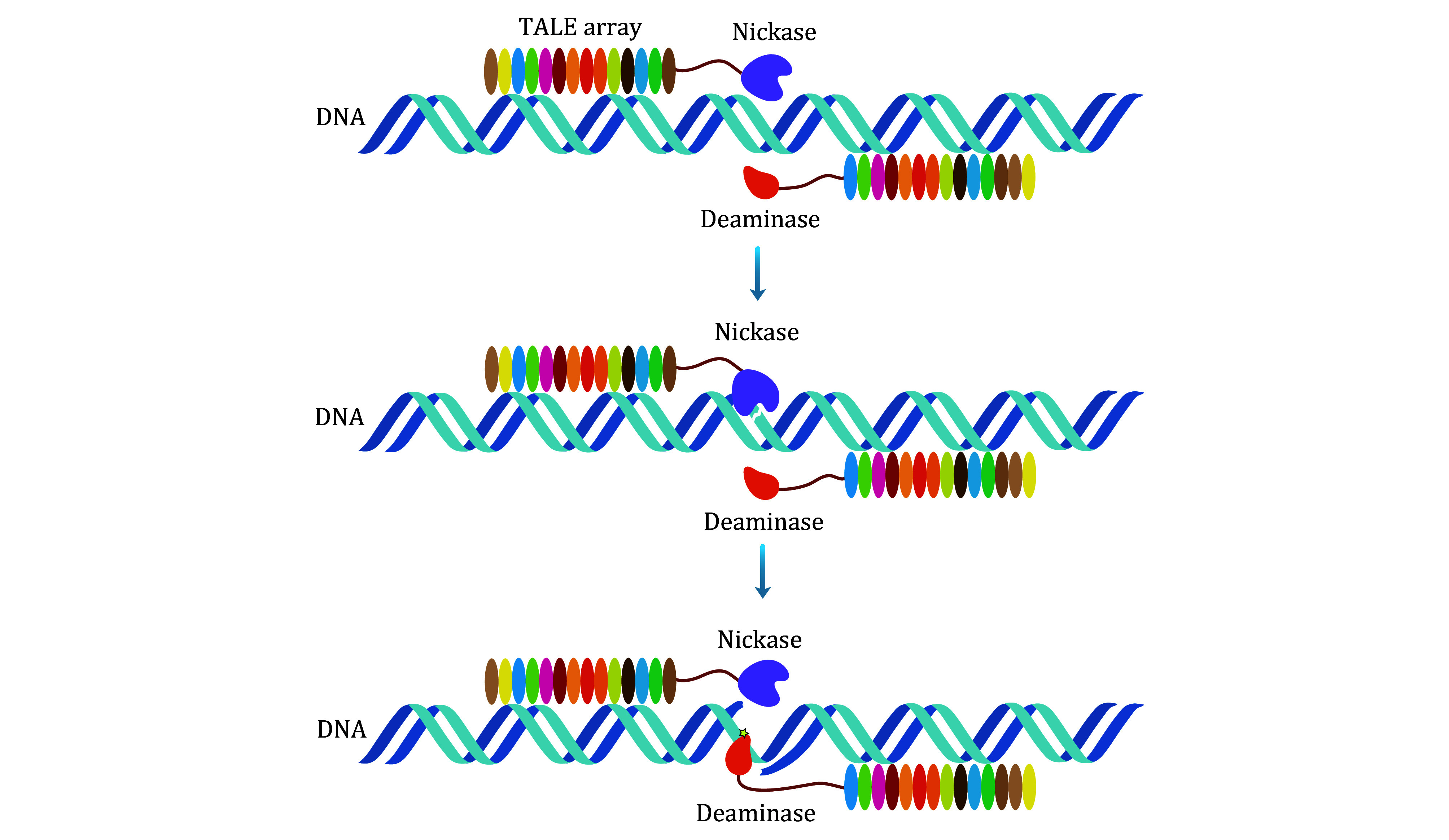
A hypothetical operational framework of mitoBEs. The TALE-nickase engages with the target DNA, inducing nicking in the dsDNA. The nicked dsDNA is likely to adopt single-stranded DNA structures. Subsequently, TALE-ssDNA deaminase interacts with the target DNA, effectively deaminating the target bases within the resulting ssDNA

To enhance the efficiency and precision of mitochondrial base editing using single-stranded DNA deaminase, the incorporation of a nickase was considered to induce the generation of single-stranded DNA. This approach involves fusing the nickase and single-stranded DNA deaminase with TALE proteins bearing mitochondrial targeting sequence to achieve mitochondrial base editing. Several nickases, including MutH (5′-↓GATC-3′), MutH(E91A, F94A) (5′-↓GAT-3′), and Nt.BspD6I(C), were identified for their high nicking efficiency. With efficient single-stranded DNA adenine deaminase (TadA8e-V106W) and cytosine deaminase (APOBEC1), this led to the development of novel mitochondrial base editing tools called mitoBEs, capable of achieving efficient and strand-selective mitochondrial base editing (Fig. 1) (Yi *et al*. 2023; Zhang and Wei 2024).

**Figure 2:**
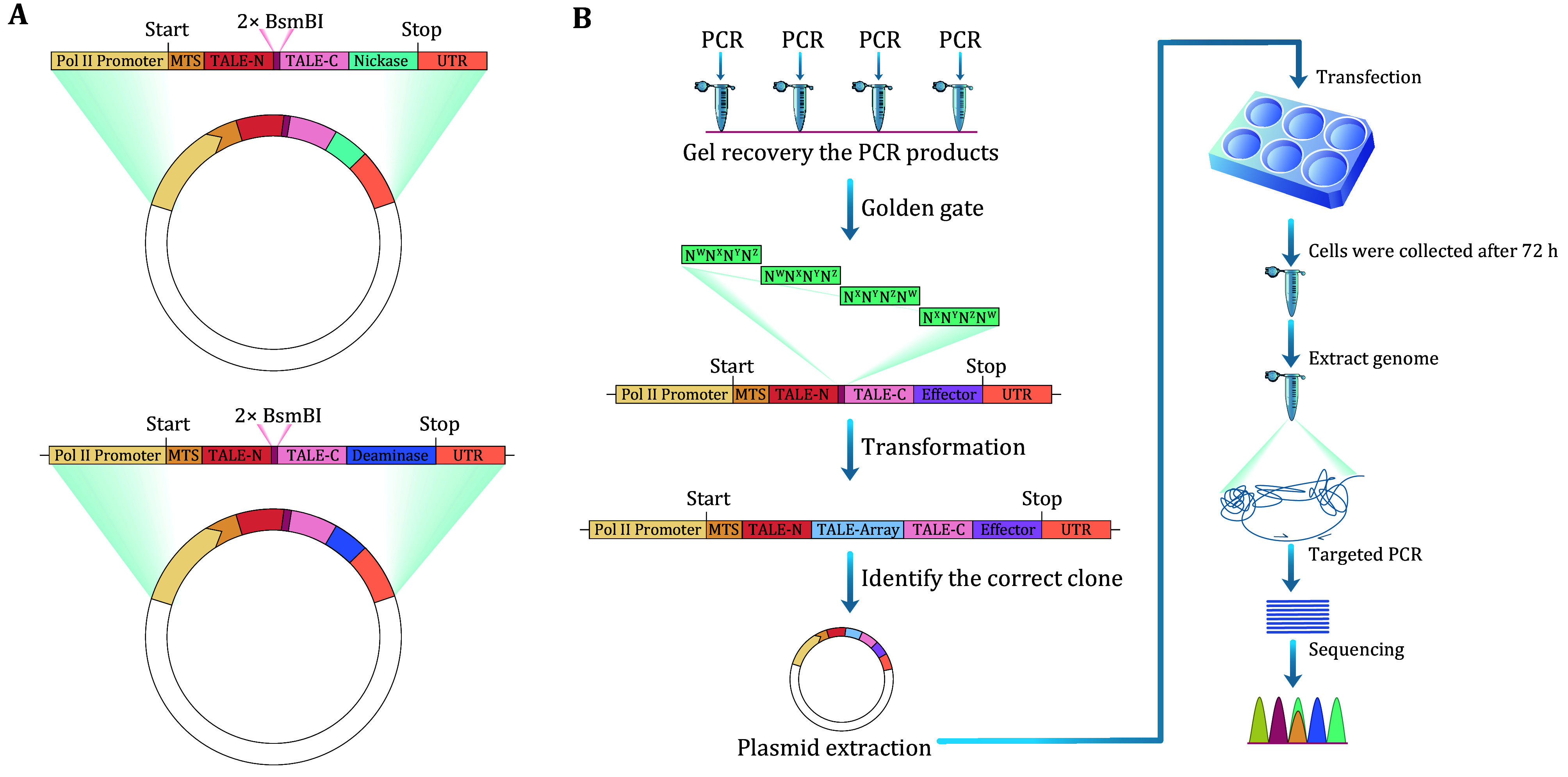
**A** Diagram illustrating TALE-preassembled mitoBEs plasmid, devoid of the TALE array sequence insertion. **B** Sequence of events in mitoBEs workflow, encompassing TALE assembly, plasmid extraction, cell transfection, genome extraction, and sequencing

In this context, we furnish a detailed experimental protocol for mitoBEs with the aim of facilitating proficient mitochondrial base editing. The process primarily encompasses the following steps: plasmid construction, circular RNA preparation (optional), cell transfection, editing efficiency detection and data analysis, and off-target detection and data analysis (Fig. 2).

## MATERIALS AND EQUIPMENT

### Reagents and supplies

• PrimeSTAR^®^ GXL DNA Polymerase (TaKaRa, cat. no. R050B)

• Gibson Assembly^®^ Master Mix (New England Biolabs, cat. no. E2611L)

• NEBridge^®^ Golden Gate Assembly Mix (BsmBI-v2) (New England Biolabs, cat. no. E1601)

• Zymoclean^TM^ Gel DNA Recovery Kit (Zymo Research, cat. no. D4008)

• DNA Clean & Concentrator^TM^-25 (Zymo Research, cat. no. D4034)

• RNA Clean & Concentrator^TM^-25 (Zymo Research, cat. no. R1018)

• EndoFree Mini Plasmid Kit II (TIANGEN, cat. no. DP118-02)

• EndoFree Plasmid Maxi Kit (QIAGEN, cat. no. 12362)

• NheI-HF (New England Biolabs, cat. no. R3131M)

• SpeI-HF (New England Biolabs, cat. no. R3133M)

• AgeI-HF (New England Biolabs, cat. no. R3552L)

• PmeI (New England Biolabs, cat. no. R0560L)

• HiScribe^®^ T7 High Yield RNA Synthesis Kit (New England Biolabs, cat. no. E2040S)

• DNase I (RNase-free) (New England Biolabs, cat. no. M0303S)

• RNase R (Epicentre, cat. no. RNR07250)

• HEK293T cell line (ATCC, cat. no. CRL-3216)

• Dulbecco's Modified Eagle Medium (Corning, cat. no. 10-013-CV)

• GlutaMAX^TM^ supplement (100×) (Gibco, cat. no. 35050-061)

• Penicillin-Streptomycin Solution (Sigma-Aldrich, cat. no. V900929)

• Trypsin (Sigma-Aldrich, cat. no. T2600000)

• Fetal Bovine Serum (Biological Industries, cat. no. 04-001-1ACS)

• PEI (Proteintech, cat. no. PR40001)

• Lipofectamine™ MessengerMAX Reagent (Invitrogen, cat. no. LMRNA008)

• P3 Primary Cell 4D-Nucleofector^®^ X Kit L (Lonza, cat. no. V4XP-3024)

• DNeasy^®^ Blood & Tissue Kit (QIAGEN, cat. no. 69506)

• Agencourt Ampure XP beads (Beckman Coulter, cat. no. 000130)

• VAHTS^®^ Multiplex Oligoes Set 4/5 for Illumina (Vazyme, cat. no. N321/N322)

• VAHTS^®^ Universal DNA Library Prep Kit for Illumina V3 (Vazyme, cat. no. ND607-02)

• VAHTS^®^ Universal Plus DNA Library Prep Kit for Illumina (Vazyme, cat. no. ND617)

### Equipment

• QIAcube (QIAGEN GmbH, Germany)

• 4D-Nucleofector (Lonza, Switzerland)

### Software

• R (version 3.6.3), R Core Team

• fastqc (version 0.11.9), https://github.com/s-andrews/FastQC

• fastp (version 0.20.1) (Chen *et al*. 2018)

• bwa-mem2 (version 2.2.1) (Vasimuddin *et al*. 2019)

• SAMtools (version 1.1) (Li *et al*. 2009)

• GATK (version 4.3.0.0) (Van der Auwera *et al*. 2013)

• HOMER (Heinz *et al*. 2010)

• MEME (version 5.0.5) (Bailey *et al*. 2015)

• BEDtools (version 2.30.0) (Quinlan 2014)

• sambamba (version 0.6.6) (Tarasov *et al*. 2015)

## PROCEDURES

### Step 1: Construct mitoBEs expression plasmids

#### Step 1.1: Construct TALE-preassembled mitoBEs expression plasmids [TIMING 4 d]

Step 1.1.1: Optimize and synthesize deaminases (*e*.*g*., APOBEC1, TadA8e-V106W), nickases (*e*.*g*., MutH and its mutant, Nt.BspD6I(C)), and uracil glycosylase inhibitor (UGI) for mammalian expression (Tsingke Biological Technology). Refer to supplementary Table S1 for corresponding sequences.

Step 1.1.2: Assemble the mitoBEs expression element into the ampicillin-resistant pCMV vector. This element includes the CMV promoter, mitochondrial targeting sequence (SOD2 or COX8 MTS), tag protein (3× HA or 3× Flag), N-terminal non-repetitive sequence of TALE, TALE array (the TALE array was replaced with two inverted BsmBI restriction sites), C-terminal non-repetitive sequence of TALE, deaminase and/or nickase, and UTR (SOD2 UTR or ATP5B UTR). See supplementary Table S2 for DNA sequences. Utilize PrimeSTAR GXL DNA Polymerase (TaKaRa) and Gibson Assembly Master Mix (New England Biolabs) for PCR and assembly, respectively (Fig. 2A).

Step 1.1.3: Transform the ligated plasmid into bacteria (*e*.*g*., *E. coli*) using heat shock.

Step 1.1.4: Culture transformed bacteria on agar plates containing ampicillin overnight.

**[TIP]** For plasmids containing APOBEC1, preferentially use BL21(DE3) pLysS Chemically Competent Cells for transformation.

Step 1.1.5: Select bacteria from a single colony and validate plasmid accuracy through Sanger sequencing.

Step 1.1.6: Purify final plasmids using EndoFree Mini Plasmid Kit II (TianGen).

#### Step 1.2: Assemble customized TALE into TALE-preassembled mitoBEs expression plasmids [TIMING 4 d]

Step 1.2.1: Determine the target editing site and select TALE recognition sequences on both sides of the target site. For efficient and precise editing, set an editing window of 15 to 18 bp, placing the target site in the middle.

**[TIP]** The TALE recognition sequence spans 15–20 bp, promoting optimal binding between the TALE protein and DNA. It is advised to utilize an editing window of 15–18 base pairs, with placing the target site at the center enhancing efficient editing. Nevertheless, varying editing sites yield different effects. For a specific target site, consider designing multiple TALE pairs or positioning the target within different regions of the editing window for experimentation. Additionally, the reduction of bystander editing can be achieved by minimizing the editing window size.

Step 1.2.2: Assemble the TALE array using the ULtiMATE system (Yang *et al*. 2013, 2016; Liu *et al*. 2020). Amplify TALE recognition sequences with specific primers (PrimeSTAR GXL DNA Polymerase), then assemble fragments into the two inverted BsmBI restriction sites using Golden Gate Assembly Mix (BsmBI-v2) (New England Biolabs) (Fig. 2B).

Steps 1.2.3 to 1.2.5: Repeat Steps 1.1.3 to 1.1.5.

Step 1.2.6: Prepare final plasmids using EndoFree Mini Plasmid Kit II (TianGen) for cell transfection.

### Step 2: Construct circular RNA-encoded mitoBEs plasmids and prepare circular RNA *in vitro*

This step is optional for cells that are difficult to transfect with plasmids.

#### Step 2.1: Construct circular RNA-encoded mitoBEs plasmids [TIMING 4 d]

Step 2.1.1: Amplify 5' arm sequence, CVB3 IRES sequence, and 3' arm sequence using PrimeSTAR GXL DNA Polymerase. Refer to supplementary Table S2 for DNA sequences.

**Figure 3:**
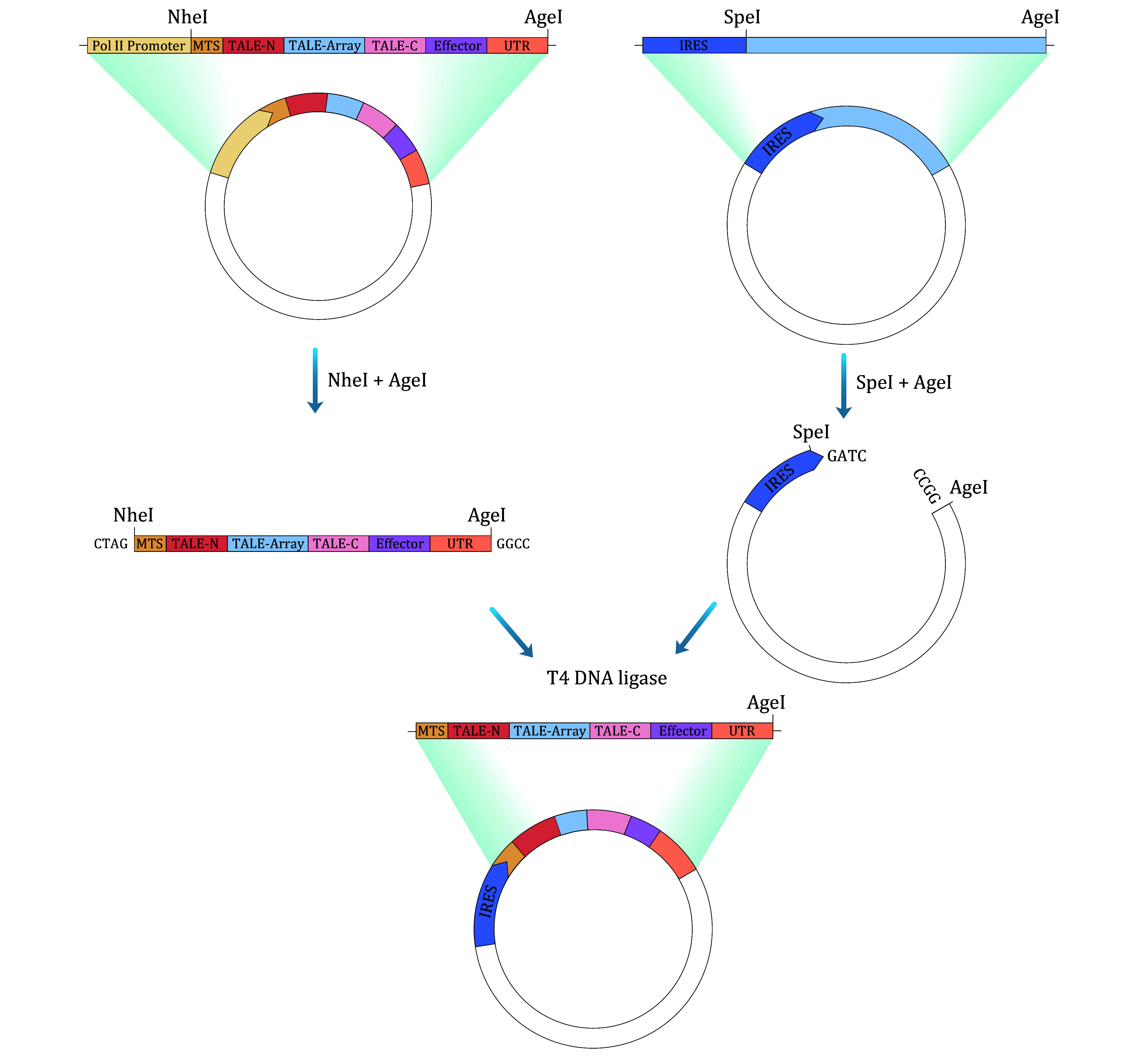
Illustration depicting the construction of mitoBEs circular RNA expression plasmid. The process involves acquiring the protein coding fragment from the assembled mitoBEs plasmid and circular RNA expression vector using restriction endonucleases, followed by ligation using T4 DNA ligase to yield the mitoBE-expressing circular RNA plasmid

Step 2.1.2: Clone fragments into a plasmid backbone via Gibson assembly to generate the empty circRNA-EV backbone. Use SpeI and AgeI cleavage sites to insert the mitoBEs coding sequence (supplementary Table S2) through a double enzyme cutting and ligation process (Fig. 3).

Steps 2.1.3 to 2.1.4: Repeat Steps 1.1.3 to 1.1.5.

Step 2.1.5: Prepare final plasmids (QIAGEN) for circular RNA preparation.

**[TIP]** Use EndoFree Plasmid Maxi Kit (QIAGEN) to avoid RNase contamination during circRNA preparation.

#### Step 2.2: Prepare circular RNA-encoded mitoBEs in vitro [TIMING 9 h]

Step 2.2.1: Linearize plasmids by digesting with PmeI for 2 h. Purify using DNA Clean & Concentrator-25 (Zymo Research).

Step 2.2.2: Synthesize circRNA precursors via *in vitro* transcription from linearized circRNA plasmid templates (1 μg) using HiScribe T7 High Yield RNA Synthesis Kit for 4 h (New England Biolabs).

Step 2.2.3: Treat the reaction products with DNase I (New England Biolabs) for 30 min to digest DNA templates.

Step 2.2.4: Add GTP to the reaction for circRNA cyclization at a final concentration of 2 mmol/L. Incubate at 55 °C for 15 min.

Step 2.2.5: Column purify RNA with RNA Clean & Concentrator-25 (Zymo Research).

Step 2.2.6: Heat-treated RNA at 65 °C for 3 min, followed by RNase R (Epicentre) at 37 °C for 15–30 min. Column purify again to obtain circular RNA.

**[TIP]** Check the final circular RNA product on an agarose gel for size and integrity.

### Step 3: Transfect mitoBEs plasmids or circular RNAs into mammalian cells

#### Step 3.1: Transfect mitoBEs plasmids into mammalian cells [TIMING 3 d]

Step 3.1.1: Using HEK293T cell as a model, distribute 7 × 10^5^ cells evenly into a 6-well plate one day prior.

Step 3.1.2: Combine the prepared plasmid (8 μg) with 200 μL Opti-MEM, and mix 16 μL PEI.

Step 3.1.3: Thoroughly blend the liquid mentioned above and let it stand at room temperature for 15 min.

Step 3.1.4: Add the mixed liquid dropwise to the 6-well plate.

Step 3.1.5: Change the culture medium 6 h after transfection to ensure cell viability.

Step 3.1.6: After 72 h, harvest cells to assess editing efficiency (Fig. 2B).

#### Step 3.2: Transfect mitoBEs-expressing circular RNA into mammalian cells [TIMING 3 d]

This step is optional and is typically performed on cells challenging to transfect with plasmids.

Step 3.2.1: Following the example of HEK293T cells, distribute 7 × 10^5^ cells evenly into a 6-well plate one day in advance.

Step 3.2.2: Combine the prepared mitoBEs-expressing circular RNA (16 μg) with Opti-MEM. Also, mix the corresponding volume (usually 1.5 times the plasmid mass, 24 μL) of Lipofectamine MessengerMAX Reagent with Opti-MEM.

Step 3.2.3: Thoroughly mix the two liquids mentioned above and let them stand at room temperature for 5 min.

Step 3.2.4: Add the mixed liquid dropwise to the 6-well plate.

Step 3.2.5: Change the culture medium 6 h after transfection to ensure cell viability.

Step 3.2.6: After 72 h, gather cells to assess editing efficiency.

#### Step 3.3: Nucleofection of mitoBEs plasmids into mammalian cells [TIMING 3 d]

This step is optional and is generally performed on cells challenging to transfect with plasmids.

Step 3.3.1: Using GM10742 cells as an example, collect 2 × 10^6^ cells and wash them with DPBS (Dulbecco’s phosphate-buffered saline).

Step 3.3.2: Combine the prepared mitoBEs-expressing circular RNA (5 μg of each plasmid) with 18 μL of nucleofector supplement and 82 μL of nucleofector solution mix (P3 Primary Cell 4D-Nucleofector X Kit L).

Step 3.3.3: Resuspend GM10742 cells in the above mix and transfer them into a nucleocuvette strip.

Step 3.3.4: Place the nucleocuvette strip into the retainer of the 4D-Nucleofector, and initiate nucleofection with the DN-100 program.

Step 3.3.5: After 72 h, collect cells to assess editing efficiency.

### Step 4: Cell collection and genome extraction [TIMING 1 h]

Step 4.1: Remove the culture medium from transfected cells, and gently wash the cells with PBS.

Step 4.2: Treat the transfected cells with 0.25% trypsin, using 100 μL of trypsin for the cells in each wall of the 6-well plate.

Step 4.3: Upon cell detachment, add 500 μL of DMEM (containing 10% FBS) medium to each well to halt digestion.

Step 4.4: Transfer the liquid containing cells to a 1.5 mL tube.

Step 4.5: Centrifuge at 2000 r/min for 5 min and discard the supernatant.

Step 4.6: Employ the DNeasy Blood & Tissue Kit for genome extraction.

### Step 5: Editing efficiency detection and data analysis

#### Step 5.1: Editing efficiency detection [TIMING 1 d]

Step 5.1.1: Design primers for deep sequencing, ensuring the forward primer is 100–150 bp upstream of the targeting site and the reverse primer is 100–150 bp downstream. Aim for a PCR amplicon length of about 200 bp.

Step 5.1.2: Perform PCR amplification following the instructions for PrimeSTAR GXL DNA Polymerase.

Step 5.1.3: Purify amplicons using the DNA Clean & Concentrator-25 kit.

Step 5.1.4: Analyze PCR products on a 2% agarose gel to identify the size of the PCR product. Redesign primers and repeat PCR amplification if no bands are observed.

Step 5.1.5: Construct targeted deep sequencing libraries using the VAHTS Universal DNA Library Prep Kit for Illumina v.3. This involves sequential steps of end repair, adapter ligation, and PCR amplification using Agencourt Ampure XP beads. The library amplification was performed using Q5U Hot Start High-Fidelity DNA Polymerase and VAHTS Multiplex Oligos Set 4/5 for Illumina. Quantify the final library with the Qubit dsDNA HS Assay Kit and sequenced using Illumina HiSeq X Ten.

#### Step 5.2: Data analysis [TIMING 1 h]

Step 5.2.1: Generate indices using BWA (v.0.7.10-r789) for the mitochondrial genome.

Step 5.2.2: Align and quantify reads using BWA (v.0.7.10-r789), then sort the BAM alignment files with SAMtools (v.1.1).

Step 5.2.3: Call variants with REDitools (v.1.0.4) using specified parameters. considered edits made by the mitoBEs with significant base conversions in targeted regions (Fisher’s exact test, *p* < 0.05).

Step 5.2.4: Export analysis results to EXCEL for further data analysis.

### Step 6: Off-target detection and data analysis

#### Step 6.1: Off-target detection [TIMING 3 h]

Input 500–1000 ng of genomic DNA for library preparation with the VAHTS Universal Plus DNA Library Prep Kit for Illumina (Vazyme). The library preparation process involves fragmentation, end preparation, dA tailing, adapter ligation, and library amplification. A mass of 500–1000 ng of genomic DNA was fragmented with FEA (Fragmentation, End Preparation & dA-Tailing) enzyme mix at 37 °C for 10 min, and end repair and dA-tailing were simultaneously completed in the process. Quantify the final library using the Qubit dsDNA HS Assay Kit (Invitrogen) and fragment analyzer, and sequenced using Illumina HiSeq X Ten (Illumina).

#### Step 6.2: Data analysis

Utilize GATK for detecting somatic mutations. It's crucial to note that GATK updates are frequent, so it is recommended to consult the most recent guidelines on the official website. To efficiency, utilize multi-threading and allocate high memory resources during alignment and mutation detection.

##### Step 6.2.1: Data preprocessing [TIMING: hours to days per RAW file]

(1) Perform basic quality control using FastQC to assess sequencing quality. Subsequently, employ fastp to eliminate adapters and bases with low sequencing quality, employing the parameter set as -q 30. Inspect sequencing depth using sambamba, setting the scratch window size to 100,000 for coverage calculation, and discard a few extremely high values.

(2) Align the data to the genome using bwa-mem2 to transform the resulting sam file to sorted bam files with samtools.

(3) Before initiating mutation searches, further process the data. Start by using GATK AddOrReplaceReadGroups to append read groups to the bam file, specifying the sequencing library, lane, and sample name. Subsequently, utilize GATK MarkDuplicates to label duplicates in the sequencing data. Apply GATK BaseRecalibrator and ApplyBQSR to adjust base quality scores based on database mutation information. If focusing solely on mitochondrial mutations, this step can be omitted.

##### Step 6.2.2: Identification of high-confidence editing sites [TIMING: hours to days per RAW file]

The objective of this module is to distinguish between editing events and spontaneous mutations in cell lines. Editing events should exhibit higher sequencing and alignment quality and be nearly absent in the control group. Conversely, their frequency should be higher in the experimental group with significant differences between the two groups.

(1) Employ GATK's Mutect2 to detect mutations from the bam files produced in the previous step. This step demands substantial computational memory and time. To alleviate these issues: firstly, compute per chromosome and then merge using MergeVcfs; secondly, increase the -native-pair-hmm-threads count, as this part in GATK supports multi-threading.

(2) Genomes inherently contain noise, and cell lines possess some heterogeneity. Thus, it is essential to further filter GATK’s VCF files. For experimental groups, set conditions for MBQ (Median base quality) and MMQ (Median mapping quality). Employ FilterMutectCalls for additional filtering. Remove sites annotated as position, slippage, weak evidence, or map qual. For control groups, ensure that the proportion of unmutated reads exceeds 99%. Conduct further filtering by calculating the *p*-value via the Mann–Whitney U-test, retaining significant (*p* < 0.1) mutations between the experimental and control groups. The last two filtering criteria draw reference from other published articles (Chen *et al*. 2023; Gaudelli *et al*. 2020; Grunewald *et al*. 2019; Lee *et al*. 2023; Sakata *et al*. 2020).

(3) Use R for graphical representation of results.

##### Step 6.2.3: Analysis of sequence features of editing sites [TIMING: minutes to hours]

For the high-confidence sites obtained in the preceding step, conduct further analysis of their features, such as examining nearby sequences resembling the used TALEN, and determining if specific motifs can be enriched.

(1) Employ bowtie2 to locate sequences in the genome similar to TALEN, setting parameters to -L 3, -p 4, -D 20, -R 3, and -a. Use BEDtools intersect to check if the expanded site coordinates overlap with similar TALEN sequence coordinates.

(2) Perform motif analysis with HOMER and MEME.

## Conflict of interest

Xiaoxue Zhang, Zongyi Yi, Wei Tang and Wensheng Wei declare that they have no conflict of interest.
